# An Atypical Case of Extrapulmonary Sarcoidosis

**DOI:** 10.7759/cureus.32164

**Published:** 2022-12-03

**Authors:** Likhita Shaik, Madeleine Wagner Sherer, Michael T Rhodes

**Affiliations:** 1 Family Medicine, Hennepin Healthcare, Minneapolis, USA; 2 Medicine, University of Minnesota School of Medicine, Minneapolis, USA; 3 Internal Medicine, Hennepin Healthcare, Minneapolis, USA

**Keywords:** atypical sarcoid, genitourinary sarcoid, osseous sarcoid, pulmonary sarcoid, extra pulmonary manifestations of sarcoidosis

## Abstract

Sarcoidosis is an idiopathic, inflammatory condition that affects nearly all organs in the body. Lungs are the most frequent and among the earliest sites for detecting it. The most common extrapulmonary manifestations involve the ophthalmic, cardiac, nervous, reticuloendothelial, cutaneous, hepatosplenic, and renal systems. These extrapulmonary manifestations of sarcoid may be misdiagnosed in the absence of the classical pulmonary features, given the high overlap of features with other chronic immunologic diseases. The diagnostic workup to differentiate sarcoid from other similar conditions is extensive, amongst which histology remains a gold standard tool for the diagnosis. Our patient presented with a chronic history of multiple vague complaints including nausea, vomiting, progressive malaise, vision changes, and weight loss. After extensive workup, a diagnosis of sarcoidosis along with multiple rare extrapulmonary involvements was made. The authors highlight essential implications including primary practice goals to avoid misdiagnosis or missed sarcoid diagnoses thus helping improve clinical outcomes in similar populations.

## Introduction

Sarcoidosis is an immune-mediated, multisystemic granulomatous disease that is pathologically represented by non-caseating granulomas. It often presents with pulmonary (dyspnea, dry cough, chest pain) and constitutional symptoms (unintentional weight loss, fatigue, night sweats) [1]. Isolated extrapulmonary manifestations (EM) of sarcoid often pose a diagnostic challenge and carry a high chance of misdiagnosis owing to sarcoid’s ability to mimic other diseases like malignancy, infections, and chronic inflammatory conditions. Among the various EM, urogenital disease and osseous are rare [2]. Urogenital sarcoid occurs in 0.2% of clinically diagnosed cases and 5% of those diagnosed at autopsy [2,3]. Osseous sarcoidosis is another rare EM with a prevalence of 3% to 13% [2-5]. It is often underdiagnosed because of its asymptomatic nature. However, early diagnosis is the key to preventing severe debilitating bone diseases such as arthritis, and complications such as spinal cord compression. We present an atypical presentation of sarcoidosis with testicular and osseous involvement [2].

## Case presentation

The patient is a 54-year-old African male who presented with a three-week history of nausea, vomiting, and constipation. Upon further interviewing, he also reported malaise, multiple episodes of photophobia, decreased vision, and a 100 lb unintentional weight loss within the past year. He denied headache, eye pain, night sweats, chest pain, dyspnea, hemoptysis, abdominal pain, lymphadenopathy, skin changes, and arthralgias. His past medical history revealed very minimal information given the lack of primary care for many years of his life. Three months prior to presentation, he was evaluated at external urgent care for an acute episode of unilateral facial numbness along the trigeminal nerve distribution.

**Figure 1:**
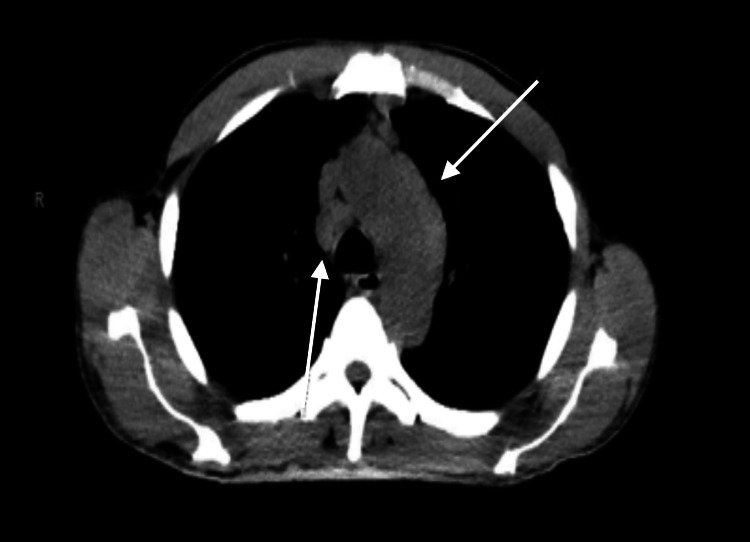
Computed tomography of the chest indicating bilateral hilar lymphadenopathy

**Figure 2:**
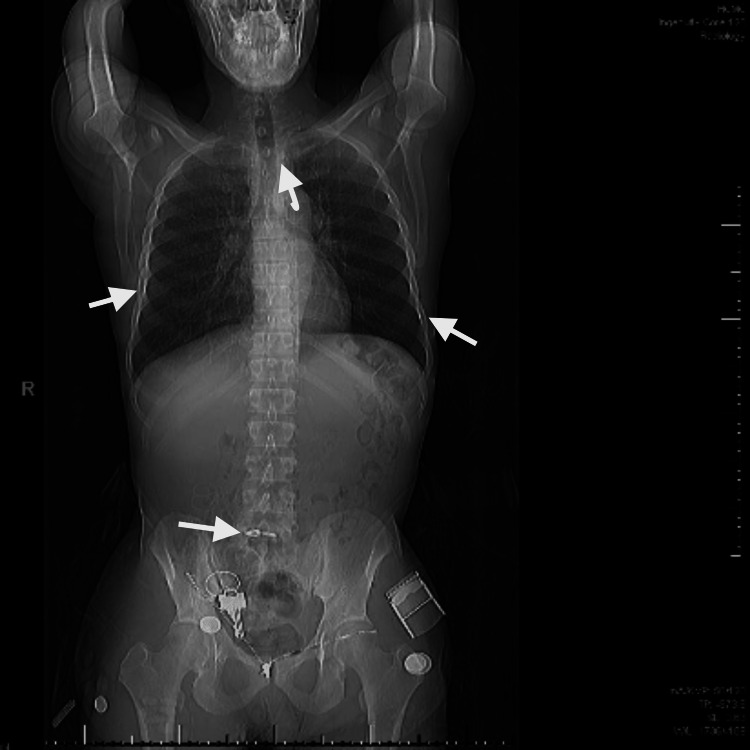
Scout scan of computed tomography imaging showing diffuse skeletal sclerosis

On arrival, vital signs were blood pressure 147/97 mm Hg, heart rate 88 beats per minute, respiratory rate 16, oxygen saturation 97% (on room air), and temperature 36.6 C. Physical examination was significant for cachexia with bitemporal wasting, skin tenting, and sunken eyes. No other muscular or subcutaneous cachexia-related findings were observed. The chest, abdomen, and neurological exams were unremarkable. Palpable lymphadenopathy was absent. Laboratory findings were consistent with normocytic anemia, acute kidney injury, hypercalcemia, and hypernatremia (Table 1). All other laboratory values were within normal limits. Nutrition-related laboratory findings showed elevated uric acid and low transferrin. Other nutrition values including inflammatory markers, such as C-reactive protein (CRP), white blood cell counts (WBC), and serum albumin were unremarkable. Non-contrast CT of the head, neck, and chest revealed mediastinal, hilar lymphadenopathy, diffuse skeletal sclerosis, and multiple lytic bone lesions (Figures 1, 2) which were suspicious for inflammatory and malignant processes. 

**Table 1:** Laboratory values

Lab	Result	Reference Range
Hemoglobin	12.9 g/L	13.1-17.5g/L
Mean corpuscular volume	86.9 fl	80.0-100.0 fl
Serum creatinine	7.1 mg/dL	0.70-1.25 mg/dL
Alkaline phosphatase	265 IU/L	40.0-129.0 IU/L
Serum calcium (pH corrected ionized calcium )	7.51 mg/dL	4.40-5.20 mg/dL
Serum sodium	152 mEq/L	135-148 mEq/L
Serum magnesium	3.2mg/dL	1.6-2.6 mg/dL
Serum phosphorous	6.5mg/dL	2.5-4.5mg/dL
Serum uric acid	12.6 mg/dL	3.4-7 mg/dL
Serum transferrin	136 mg/dL	200-360 mg/dL

**Figure 3:**
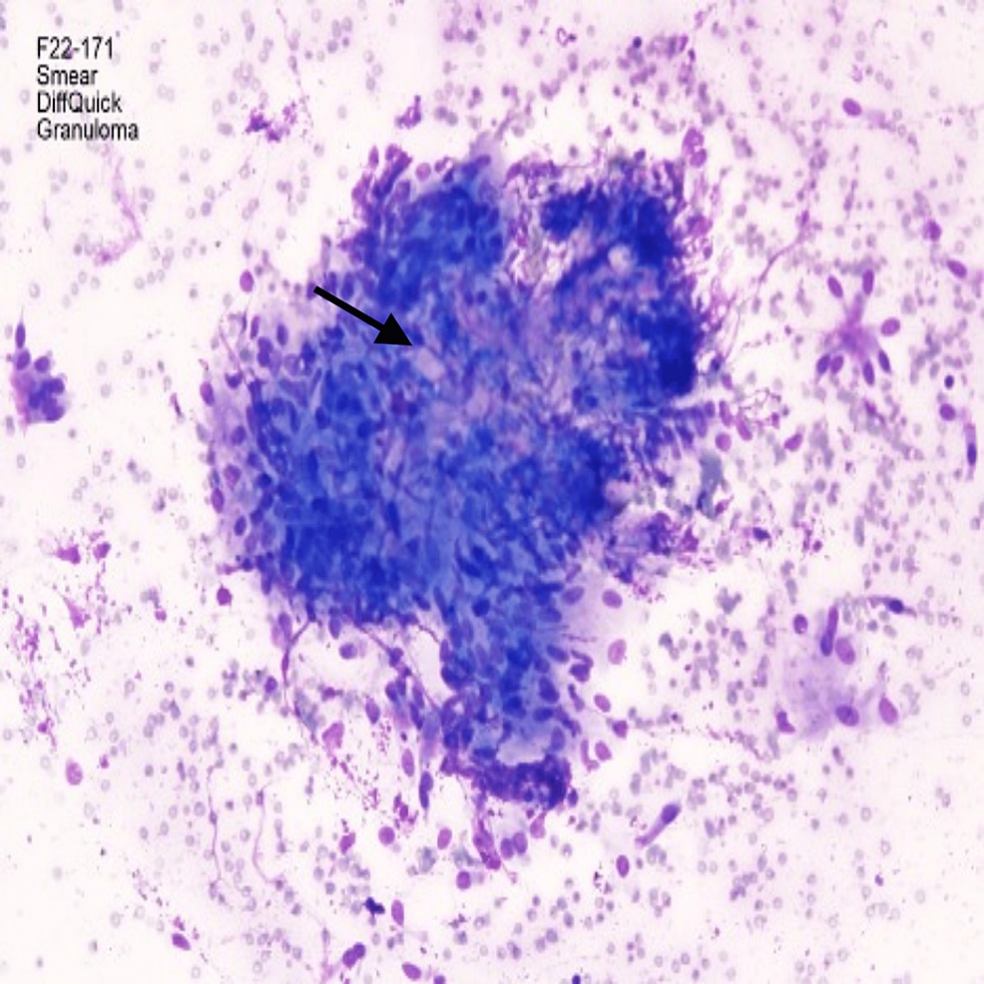
Pathology from fine needle aspiration (FNA) of subcarinal lymph node obtained with endobronchial ultrasound (EBUS) showing epithelioid histiocytes in a pattern consistent with non-necrotizing granulomatous inflammation. Stain: Diff-Quick; Magnification: 200x

**Figure 4:**
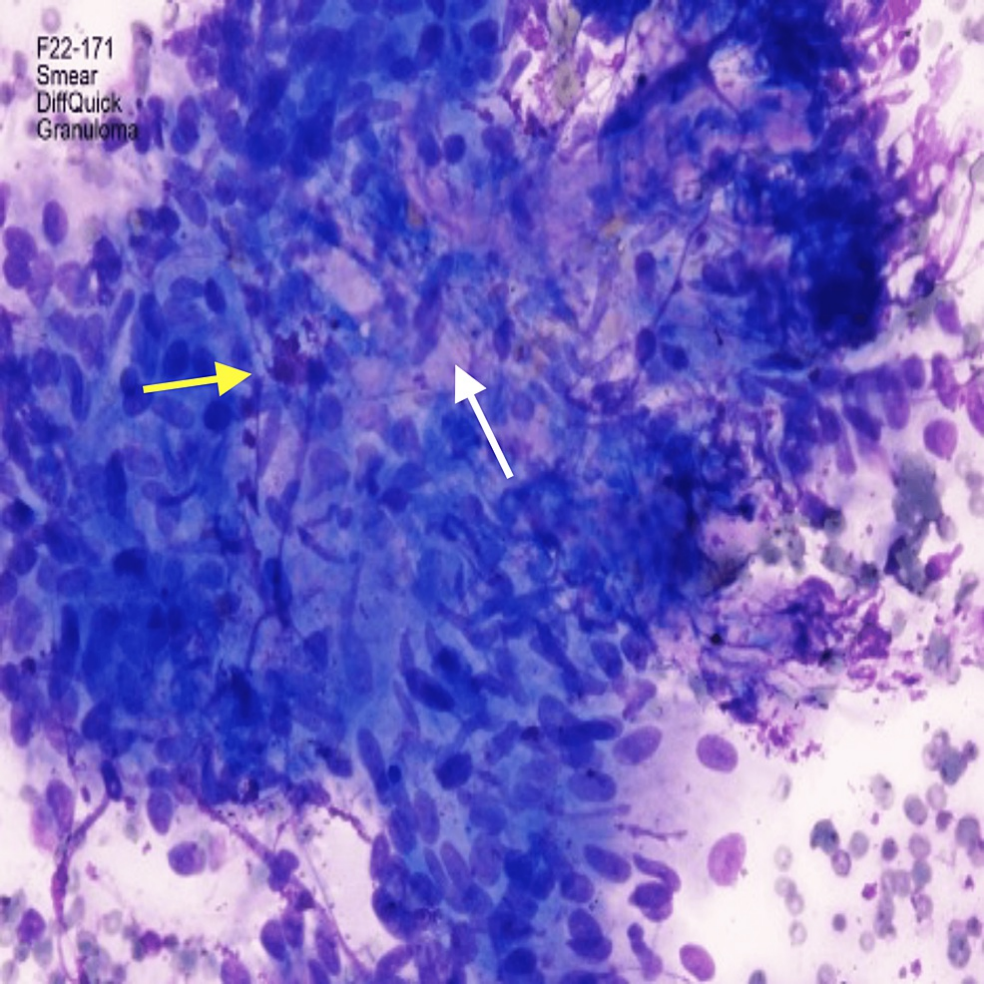
Higher magnification of pathology from fine needle aspiration (FNA) of subcarinal lymph node obtained with endobronchial ultrasound (EBUS) showing epithelioid histiocytes (yellow arrow) in a pattern consistent with non-necrotizing granulomatous inflammation (white arrow). Stain: Diff-Quick;  Magnification: 400x

**Figure 5:**
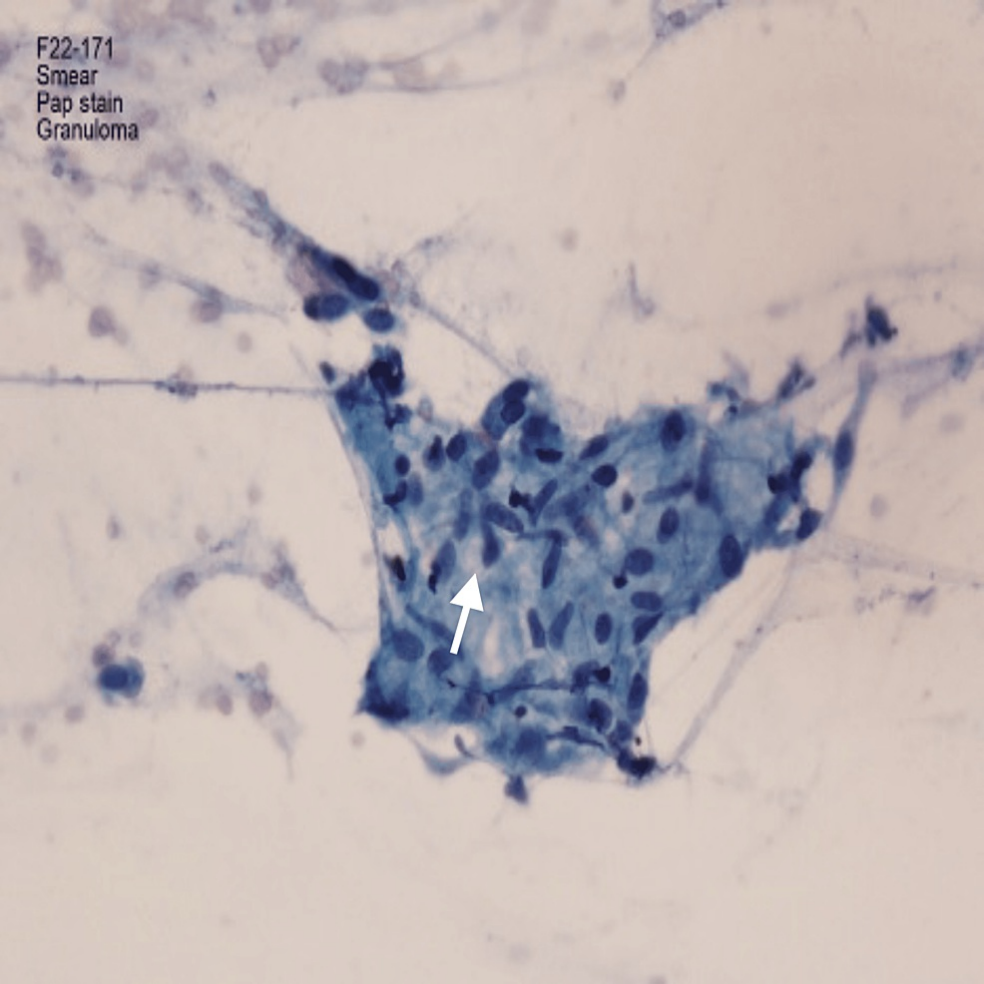
Papanicolaou-stained pathology from fine needle aspiration (FNA) of subcarinal lymph node obtained with endobronchial ultrasound (EBUS) confirming that this is not caseating necrosis, which would appear gray-green (arrow). Stain: Pap; Magnification: 400x

Given the chronicity of the patient’s presentation and imaging findings, the patient was admitted for further diagnostic workup. Multiple respiratory cultures, acid-fast bacilli (AFB) smears, and potassium hydroxide preparations were negative for any infectious pathogens. The patient tested negative for HIV. Fine needle aspiration (FNA) of an epitrochlear lymph node was non-diagnostic for which the patient underwent endobronchial ultrasound (EBUS) with FNA of hilar lymph nodes. This biopsy revealed non-caseating granulomas (Figures 3, 4, 5). Angiotensin-converting enzyme (ACE) level was later found to be elevated, consistent with the diagnosis of pulmonary sarcoidosis. After obtaining a negative Strongyloides serology, treatment with prednisone was initiated. Given the need for chronic treatment with steroids, the patient was prescribed *Pneumocystis jiroveci *prophylaxis with double-strength trimethoprim-sulfamethoxazole. The patient did not meet the criteria for peptic ulcer disease prophylaxis.

**Figure 6:**
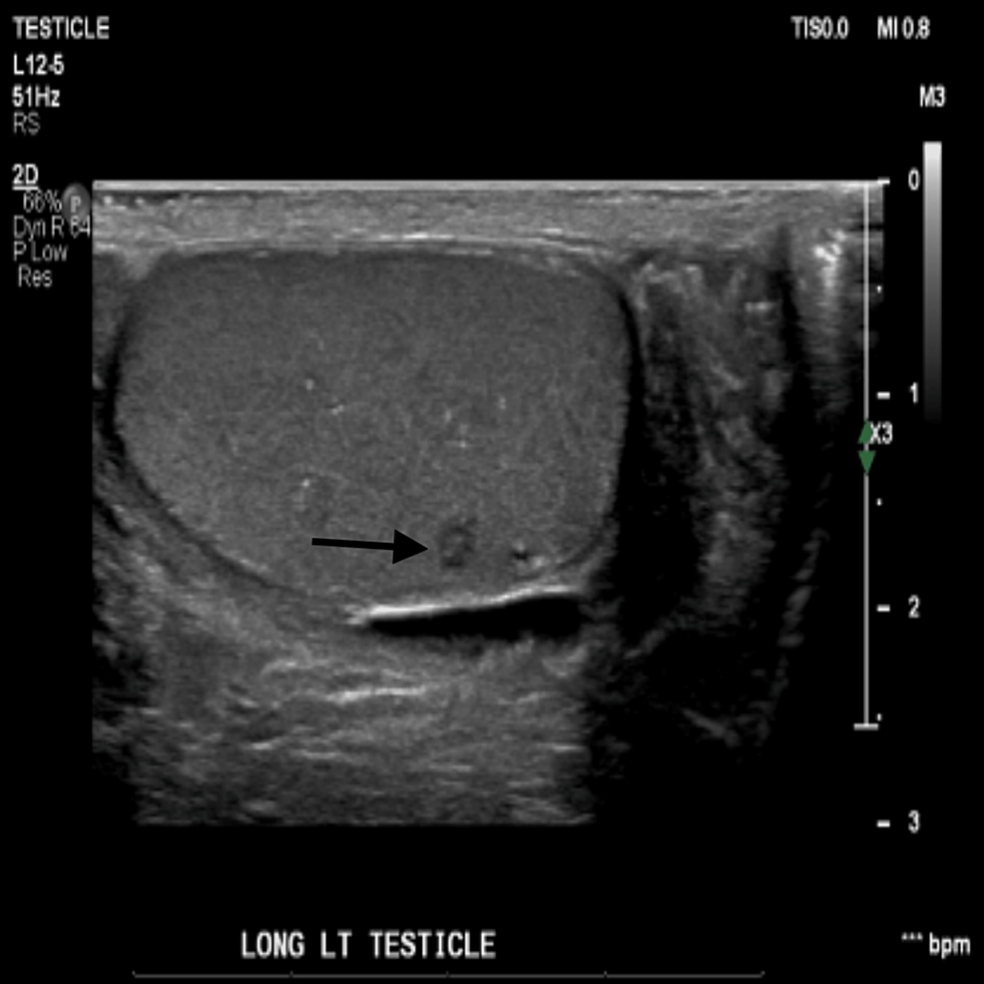
Scrotal ultrasound of the left testicle, longitudinal axis, which reveals an area of hypoechoic focus consistent with granuloma.

Further workup to identify extrapulmonary involvement was undertaken. Ophthalmology was consulted for eye involvement. Their exam showed bilateral uveitis with posterior synechiae and secondary glaucoma consistent with ocular sarcoidosis for which timolol, prednisolone, and cyclopentolate were initiated. Thyroid, barium swallow, and echocardiographic studies were unremarkable. Abdomen imaging did not show any other lesions suspicious for sarcoid. On the day of scheduled discharge, the patient complained of sharp bilateral testicular pain though with an unremarkable genitourinary exam. Given low suspicion for sexually transmitted diseases and normal urinalysis, scrotal imaging was done which revealed inflammatory changes consistent with right epididymitis and a small left testicular granuloma (Figure 6). Antibiotics were not indicated in the absence of infectious signs and the prescribed steroid course was assumed to treat the left testicular sarcoid. The patient was discharged with close pulmonology and primary care follow-up. 

## Discussion

Our patient presented with a constellation of vague symptoms (nausea, vomiting, malaise, weight loss) without specific pulmonary complaints. One of the critical findings prompting further evaluation (and thus leading to the diagnosis of sarcoidosis) was the amount of unintentional weight loss which as reported by the patient was 100 pounds in one year. An evaluation of this reported weighted loss and facial numbness at an external facility three months prior prompted non-contrast CT of the head, neck, and chest. This imaging showed bilateral hilar lymphadenopathy. However, the patient was lost to follow-up as the patient dismissed the importance of further diagnostic workup. When the patient presented at our hospital, the continued presence of bilateral hilar lymphadenopathy and other abnormal laboratory findings such as normocytic anemia, acute kidney injury, hypercalcemia, and hypernatremia along with a significant weight loss, instigated the need for further workup. Broad differential diagnoses from benign conditions to malignancies were formed. The patient needed to be educated on the need for further investigations in the current clinical scenario. Multimodality and a multidisciplinary team approach along with the availability of technology for bronchoscopic fine needle aspiration, ACE levels, AFB smears, and cultures were key resources that aided in diagnosing sarcoidosis after ruling out other diagnoses.

The diagnosis of sarcoidosis is challenged by the heterogeneous nature of the disease. There is a higher incidence of sarcoidosis in association with certain prior infections and occupational exposures, particularly those involving significant or extensive inhalation of particles, but no single trigger. Genetic background and autoimmunity may also play a role [2,6]. Patients often present with nonspecific complaints or may present primarily with extrapulmonary manifestations. There are no pathognomonic exam findings or screening laboratory tests for sarcoidosis. An elevated level of ACE has low sensitivity and specificity for sarcoidosis [1]. Furthermore, while an estimated 90% of patients with sarcoidosis have pulmonary involvement, only approximately 40% have the classic finding of bilateral hilar lymphadenopathy on chest radiography. Diagnosis of sarcoidosis relies on a combination of clinical and radiographic findings supported by histologic evidence of non-necrotizing granulomas. Final diagnosis also requires the exclusion of other possible causes, including tuberculosis, interstitial lung disease, fungal infection, lymphoproliferative disorders, and autoimmune disorders such as Sjogren syndrome [6]. 

After diagnosing sarcoidosis, evaluation for extrapulmonary manifestations is important to determine prognosis and additional treatment. The most involved systems include cardiovascular, cutaneous, ophthalmologic, and neurologic (Table 2). Cardiac sarcoidosis most often involves left ventricular arrhythmias, and congestive heart failure which are the leading causes of death in sarcoidosis. Hence, evaluation for the presence of cardiac disease is vital despite the lack of symptoms [2,7,8]. Musculoskeletal, gastrointestinal, renal, and genitourinary are among the least commonly involved systems [2,7].

**Table 2:** Clinical manifestations of sarcoidosis

Organ System	Incidence Rate	Manifestations
Pulmonary [6]	90%	Cough, Dyspnea, Chest pain
Cardiac [2]	5% with clinical manifestations 20% to 30% with granulomas found at autopsy	Arrhythmia, Palpitations, Presyncope, Syncope, Dilated cardiomyopathy, Valvular involvement (rare)
Cutaneous [2]	20% to 35%	Erythema nodosum, Papular lesions (most commonly on the face), Maculopapular lesions (most commonly on the neck and trunk), Plaque lesions (associated with chronic disease), Lupus pernio (associated with more aggressive disease)
Ophthalmologic [2]	10% to 60%	Uveitis, Ocular inflammation, Retinal vasculitis
Neurologic [2]	5%	Cranial nerve dysfunction; Peripheral neuropathy; Granulomatous involvement of brain parenchyma, cerebellum, brainstem, or leptomeninges
Musculoskeletal [2]	Osseous: 3% to 13%, Muscle involvement: 5% (clinically)	Arthralgia, Lytic, Permeative or destructive bony lesions, Bone cysts, Symmetric proximal weakness, Nodular myopathy
Gastrointestinal [2]	Liver: 50% to 80%, Pancreas: 5%, Luminal GI tract: rare	Pruritus, Jaundice, Ascites, Cirrhosis (rare), Biliary disease
Renal [2]	7% to 23%	Granulomatous interstitial nephritis, Sterile pyuria, Proteinuria, Microscopic hematuria, Hypercalcemia, Hypergammaglobulinemia
Genitourinary [9]	0.2% clinically diagnosed, 5% at autopsy	Painless or painful testicular swelling; Ureter involvement causing flank pain, hydronephrosis, hematuria; Urinary bladder involvement causing hematuria, difficulty voiding, ureteral obstruction, penile or prostate granuloma

Musculoskeletal manifestations are common and occur as various arthritic syndromes, acute myopathy, nodular myopathy, and chronic myopathy. Osseous involvement is typically associated with chronic multisystem disease progression through lytic, permeative, and destructive processes. The bones of the hands and feet are most often affected, although granulomas may also develop in the skull, long bones, ribs, pelvis, and axial skeleton. These lesions are often asymptomatic and if undiagnosed may result in severe bone damage and fractures in a long run [4,10]. Hence, even an asymptomatic bone disease warrants monitoring for bone changes. 

Granulomatous involvement of the kidney though rare can present with signs of interstitial nephritis in diffuse sarcoidosis. Our case represents an atypical form of interstitial nephritis through hypercalcemia and hypergammaglobulinemia occurring with grossly normal renal imaging in the setting of moderate extrapulmonary disease. Genitourinary disease particularly follows the course of granulomatous renal disease. This was atypical in our patient with an incidental finding of testicular granuloma with grossly normal renal imaging. Testicular sarcoidosis has been rare and substantially underdiagnosed as per the available literature with an incidence as high as 5% at autopsy. A hypoechoic lesion on B-mode ultrasound serves as a diagnostic for the testicular sarcoid. However, equivocal radiologic suspicion needs further differentiation of mimicking lesions (seminoma, Leydig cell tumor, lymphoma, and testicular cancer) by contrast-enhanced ultrasound. Moreover, the association between sarcoidosis and testicular malignancy mandates regular monitoring [3,10,11]. 

Our case highlights how vague symptoms pose as only clues to prompt further investigations for immune-mediated diseases like sarcoidosis. Like our patient’s case, primary care physicians are often the first point of care contacts for such patients. Exploring patients' sociocultural background, and particular fears and concerns help in negotiating refusal of care and the need for further workup. Supporting patients through their concerns about the idiopathic nature of this disease by addressing their doubts and questions helps navigate the orthodox perception of medical care in certain cultures [12,13]. While, underdiagnosing and undertreatment of such conditions may lead to worsening and progression of the disease, delaying the diagnosis draws an unnecessary potential to increase healthcare costs that may be out of pocket, extra office visits, unnecessary diagnostic testing, inappropriate additional treatments, and associated psychological trauma experienced by patients. Primary care physicians also play a vital role in such situations by providing resources about the unknown etiology of the disease, its prognosis, and treatment options, including the risks and benefits [14-17]. 

## Conclusions

Sarcoidosis presents with myriad vague symptoms that mimic various infectious, inflammatory, and malignant pathologies that call for exclusion before its diagnosis. A thorough history and extensive diagnostic workup should be undertaken to screen for extrapulmonary sarcoidosis. Cardiac, bone, renal and genitourinary diseases are among the most missed and dangerous EM owing to the poor localization of symptoms. Though few forms of osseous, renal, and genitourinary sarcoid occur only in diffuse sarcoidosis, atypical cases with such involvements do occur sporadically with minimal-moderate disease too, and hence should not be missed. Close vigilance by primary care physicians, guided by a multidisciplinary approach aid in the effective diagnosis of sarcoidosis. 
